# Health behavior, loneliness and social participation among students in a higher education institution: a community case study providing insights into health psychology

**DOI:** 10.3389/fpsyg.2026.1894036

**Published:** 2026-10-12

**Authors:** Sonia Lippke, Zhi Ye, Klaus Boehnke, Alexander Strelnikov, Lingling Gao

**Affiliations:** 1 Health Promotion and Prevention with Special Focus Rehabilitation Sciences, Faculty of Health, Hamburg University of Applied Sciences (HAW Hamburg), Hamburg, Germany; 2 School of Business, Social and Decision Sciences, Constructor University, Bremen, Germany; 3 Laboratory of Public Safety Risk Governance, Zhejiang Police College, Hangzhou, China; 4 Bremen International Graduate School of Social Sciences (BIGSSS), Constructor University, Bremen, Germany; 5 Department of Applied Psychology, Nantong University, Nantong, China

**Keywords:** community case study, contextual factors, health behavior, health psychology, higher education institution, loneliness, psychological resources, social participation

## Abstract

**Background:**

Higher education institutions can be conceptualized as *community settings* that may facilitate individuals’ health and well-being (in terms of low loneliness), health behavior, and general social participation. However, facilitating factors in individuals, their social network, and contextual factors were not researched building on the *Evolutionary Theory of Loneliness* and the *Compensatory Carry-Over Action Model*.

**Objective:**

In a community case study, students’ health behavior, loneliness, and social participation were investigated to determine interrelations and factors that help students maintain a healthy lifestyle, reduce loneliness, and increase social participation.

**Method:**

We analyzed an online survey of 373 students using descriptive statistics, group comparisons, regression models, and exploratory curve estimation.

**Results:**

Of 293 students with available loneliness data, the mean loneliness was *M* = 2.76 (SD = 1.06; range 1–4), and 122 (41.6%) were classified as lonely. Loneliness was reflected in private relationships and study-related contacts, whereas no significant association was found with studying/working from home, χ^2^(1) = 0.11; *p* = 0.74. Lonely students differed from not-lonely students across 10 behavioral domains, including talking to a doctor, watching films, spending time in nature, interacting with others online, listening to music, drinking alcohol, eating, and shopping. For a healthy lifestyle, the regression results highlighted psychological resources and perceived health: self-efficacy predicted the healthy lifestyle indicator in Step 1 (OR = 2.40), and health literacy responsibility was significant in Step 2 (OR = 1.78), but poor subjective health was the strongest predictor in the final model (OR = 0.52; *R*^2^ = 0.21). Loneliness was negatively associated with social participation (ß = −0.30), but this association weakened when further psychological and contextual factors were considered. Exploratory curve estimation indicated an inverted U-shaped association between stress and social participation among not-lonely students (*R*^2^ = 0.09; *p* = 0.03), whereas this pattern was not statistically significant among lonely students.

**Conclusion:**

Students’ loneliness should not be understood as social disconnection, but as part of a broader health-psychological pattern involving health and well-being, psychological resources, contextual factors, and social participation within the higher education community.

## Introduction

### General concepts of the study

*Health Psychology* is a dynamic and evolving field concerned with how psychological, behavioral, social, and environmental factors interact to shape health, well-being, and illness, including loneliness (Banerjee et al., 2023). Consistent with the biopsychosocial perspective, health outcomes cannot be fully understood without considering the interplay between individual experiences, health-related behaviors, social relationships, and contextual influences (Migliorini et al., 2026). Increasing attention has therefore been directed toward social connectedness and participation as key determinants of psychological and physical health. Within this broader framework, loneliness has emerged as a particularly important public health concern because of its associations with reduced well-being, poorer mental health, and adverse behavioral outcomes across diverse populations (Binte Mohammad Adib and Sabharwal, 2024).

### Narrowing the focus to the higher education context

This is particularly relevant in higher education institutions (HEIs), where students navigate academic, social, and cultural transition (Ellard et al., 2023; Zahedi et al., 2022). University students frequently face challenges related to academic pressure, social integration, adjustment to new environments, and the development of supportive interpersonal networks. Such challenges may be amplified in internationally diverse settings, where students often encounter additional cultural, linguistic, and social adaptation demands. Research has demonstrated that experiences of loneliness, social participation, and belonging are closely linked to students’ academic engagement, persistence, well-being, and successful adjustment to university life (Dost, 2026; Pleschová et al., 2026; Varsou and Welsh, 2026). Although these relationships have been documented across different HEIs, important questions remain about how institutional environments can effectively support social integration and psychological well-being among highly diverse student populations.

### Prior work and theoretical background

Accordingly, this community case study adopts a *biopsychosocial perspective* and focuses on psychological, interpersonal, and environmental processes that shape both mental and physical health (Smith et al., 2016). Specifically, it addresses open questions related to community health psychology, health behaviors, and intervention models targeting psychological well-being/stress perception and social participation (Cicognani et al., 2008; Knapp et al., 2024; Migliorini et al., 2026; Youn et al., 2020): Universities and HEIs represent a critical microcosm for examining these processes, offering opportunities to explore how institutional environments can promote sustainable, healthy lifestyles, reduce inequalities, and foster inclusive, supportive communities in line with global goals such as SDG 3 (Good Health and Well-being) and SDG 10 (Reduced Inequalities) (Ellard et al., 2023; Hysing et al., 2020; Södel et al., 2025).

### Evolutionary theory of loneliness

A central theoretical framework guiding this study is the *Evolutionary Theory of Loneliness* (ETL) (Cacioppo and Cacioppo, 2018). The ETL conceptualizes loneliness as an adaptive, evolutionarily grounded signal that motivates individuals to restore social connection (Lippke and Pappa, 2025b). While short-term loneliness can enhance vigilance and promote reconnection, chronic loneliness is associated with adverse psychological, behavioral, and physiological outcomes. Importantly, the ETL highlights that loneliness emerges from the interaction of personal characteristics, behavioral responses, and environmental contexts, making it highly relevant for understanding student experiences in HEIs, particularly among those navigating new cultural and social environments (Homer et al., 2026; Hysing et al., 2020). From a belonging perspective, chronic loneliness may be understood as reflecting unmet needs for meaningful social relationships and integration within valued communities, explaining why students do not perform social participation (Dost, 2026). Therefore, the ETL provides a useful framework for examining how social participation and institutional belonging jointly contribute to student health and well-being.

### Compensatory carry-over action model

To complement this perspective on health behaviors, the *Compensatory Carry-Over Action Model* (CCAM) (Lippke and DeSmet, 2025) provides a framework for understanding how multiple health-related behaviors interact and are shaped by higher-level goals. Rather than viewing behaviors such as physical activity, nutrition, social engagement, and help-seeking in isolation, the CCAM emphasizes their interdependence. Students must allocate limited cognitive, emotional, and temporal resources across competing domains, leading to compensation and carryover. For example, intensive academic engagement may reduce resources for social interaction or self-care, while success in one domain may strengthen self-efficacy and facilitate engagement in others (Lippke and DeSmet, 2025; Lippke and Pappa, 2025a,b). This dynamic interplay is crucial for understanding how loneliness relates to broader lifestyle patterns and health behaviors.

### Integrating ETL and CCAM

Integrating the ETL and CCAM perspectives enables a comprehensive understanding of student well-being as a product of interconnected emotional, behavioral, and contextual processes. *Loneliness* not only reflects unmet social needs but may also impact and be influenced by health behaviors, self-efficacy, health literacy, and access to contextual resources (Lippke and Gan, 2024). This aligns with innovative views in health psychology to move beyond single-behavior models and adopt more integrative approaches that capture the complexity of real-world health dynamics across diverse populations (Lippke and DeSmet, 2025).

### Uniqueness of the current study

The present research addresses these questions through a *community case study,* conducted at a higher education institution (HEI), characterized by a particularly high proportion of students from different countries and cultural backgrounds. Furthermore, different support systems exist, such as:

- Academic advising and mentoring systems, as well as corresponding faculty information strategies.
- Student support services.
- Psychological and intercultural counseling services.
- Explicit mental health services.
- Resident life and peer mentor support.
- Health and well-being services as well as preventive healthcare consultations.
- De-stressing activities and events such as yoga, podcasts, and club activities & events.
- Referral support with finding a medical doctor in medical support services.
- Further student health info hub and support web/internal page.

By conceptualizing this exemplary HEI as a community case informed by *community-based participatory research* (CBPR) approaches, we aim to provide the basis for a better understanding of psychological processes in the community of students and for the development or refinement of evidence-based programs and practices for widespread dissemination (Akhter-Khan and Au, 2020; Bernardon et al., 2011; Binte Mohammad Adib and Sabharwal, 2024; Ellard et al., 2023; Hickin et al., 2021; Hysing et al., 2020; Lekamge et al., 2025; Pointon-Haas et al., 2024; Sørensen et al., 2012). Therefore, this study aims to contribute to emerging themes in health psychology, particularly the integration of emotional, behavioral, and social determinants of health in community settings. It further provides applied insights into how universities can operationalize theory-informed, interdisciplinary approaches to promote well-being, strengthen social participation, and support diverse student populations within a comprehensive support ecosystem. The findings are expected to advance understanding of how universities can foster healthy, inclusive, and supportive environments for diverse student populations. In addition, the study provides practical insights for the development, refinement, and dissemination of theory-informed interventions that promote well-being, strengthen social participation, and reduce inequalities. As such, the research aligns with broader public health and sustainability objectives, including Sustainable Development Goal 3 (Good Health and Well-being) and Sustainable Development Goal 10 (Reduced Inequalities) (Ellard et al., 2023; Södel et al., 2025).

*Research questions were the following:*

Research Question 1: (a) How lonely are students feeling relating to studying/working from home, their contacts at the university, and in private life, etc.?, and (b) if students report engaging in specific activities to cope with loneliness, do they differ in their loneliness from those not engaging in these activities (such as talking to friends, family, professionals or being in solitude, doing sports, watching TV, getting into the nature, doing gardening, cooking, reading, looking for others on the Internet or social media, snacking, shopping etc.)?
Research Question 2: How healthy are students’ lifestyles, and what role does loneliness play in this (in comparison to self-efficacy, health literacy, context, and socio-demographic factors)?
Research Question 3: To what extent do health psychological factors (lifestyle, self-efficacy, health literacy, context, and mental health aspects) help students to perform social participation in their university over and above loneliness?

The following section describes how these research questions were investigated in this *community case study*.

## Materials and methods

Eligible students were recruited via the university’s general e-mail distribution list, which included a direct link to the online survey. To maximize reach, we used additional recruitment strategies: the university’s health-promoting team informed students during special events, and we distributed and displayed print materials (posters, flyers, and table displays) across campus. Furthermore, the survey was promoted through posts and stories on the university’s official social media channels.

All enrolled university students were eligible to participate in the study. *Inclusion criteria* comprised sufficient English language proficiency (reading and writing), Internet access, and current student status. Prior to participation, all individuals received detailed study information and provided informed consent electronically before accessing the questionnaire.

This community case study followed a structured, multi-phase procedure: (1) development of the online questionnaire; (2) obtaining *ethics approval and pre-registration* of the study, including clinical trial registration (ClinicalTrials.gov ID: NCT06899308); (3) distribution of the questionnaire and data collection, conducted between March and July 2025; and (4) quantitative data analysis of the collected student data. The study design was observational.

Data were collected with the online survey platform SoSci Survey, which complies with German data protection regulations. All participant-facing materials, including study information and consent forms, were developed to be clear and accessible, pilot-tested, and approved by the responsible university ethics committee. No risks, burdens, or adverse effects for participants were anticipated.

All data were processed in anonymized form (i.e., without personally identifiable information). Results were reported exclusively in an aggregated form (e.g., means and frequency distributions). Taking part in the study was voluntary and anonymous, and no incentives were provided.

Data handling complied with the university’s privacy policy and the General Data Protection Regulation (Datenschutzgrundverordnung, DSGVO). All data were treated with strict confidentiality and high security standards and were not shared with third parties. Pre-registration further ensured transparency and adherence to good scientific practice.

The *study sample* comprised university students who participated in the health surveillance 2025 survey. Participants provided self-reported data on mental health, perceived stress, life satisfaction, coping strategies, and health-related lifestyle behaviors. A total of *N* = 373 students completed at least part of the survey. Due to item non-response, sample sizes varied across analyses, ranging from *n* = 220 to *n* = 288 depending on the variables included.

*Data* were collected to assess health behavior, health-promoting contextual resources, loneliness, self-efficacy, health literacy, symptoms of anxiety and depression, subjective health status, and study satisfaction. Wherever possible, we used validated, standardized instruments. Composite variables were calculated as mean scores across items unless otherwise specified.

### Health literacy

Health literacy was assessed using parts of a validated 10-item scale (Lenartz et al., 2020). The items were worded “When I feel unwell, I usually know exactly why,” “I pay attention to my health,” “I can sense my feelings clearly,” “I think it’s important to take care of my health”, “I notice when I am physically tense in a situation,” “I consciously take responsibility for my health,” “I can perceive my own needs well.”, “I take care of my body,” “I have good access to my feelings,” and “I consciously take care of my health, e.g., by eating a balanced diet or by getting enough exercise.” Items were rated on a 4-point Likert scale ranging from 1 (“does not apply at all”) to 4 (“fully applies”), with higher scores indicating better health literacy.

### Depression and anxiety (PHQ-4)

Symptoms of depression and anxiety were measured using the 4-item Patient Health Questionnaire (PHQ-4), which is a validated scale (Cronbach’s α = 0.85) (Kroenke et al., 2009). The scale includes two items on depressive symptoms and two on anxiety symptoms. The items were assessed with the stem “How often have you felt that the following symptoms have bothered you over the last 2 weeks?” and the items were “Little interest or pleasure in doing things,” “Feeling down, depressed or hopeless,” “Feeling nervous, anxious or on edge,” and “Not being able to stop or control worrying.” Responses ranged from 1 (“not at all”) to 4 (“nearly every day”), with higher scores reflecting more psychological distress. A composite mean score was calculated across all four items.

### Higher education institution stress (HEI-Stress)

Study-related stress was measured using a 3-item HEI-Stress scale (Schmidt et al., 2019), which was validated (Cronbach’s α = 0.84–0.89) before. The items were worded “Over the past 4 weeks: How stressed did your studies make you feel? “; “How often have you felt ‘stressed and tense’ in the past 4 weeks?”; “How would you describe your life right now?”. Additionally, a single-item measure assessed general perceived stress (“How much stress do you feel in your life in general?”), using a comparable response range, with higher scores indicating higher stress. Possible responses ranged from 0 (“not at all stressed”) to 100 (“very stressed”).

### Loneliness

Loneliness was assessed using two approaches. First, a short 3-item version of the *UCLA Loneliness Scale* (Hughes et al., 2004; Russell et al., 1980) was applied, with response options ranging from 1 (“hardly ever”) to 5 (“often”), where higher scores indicate greater loneliness. This scale was validated (Cronbach’s α = 0.72), and the items are worded as follows: “How often do you feel that you lack companionship?” “How often do you feel left out?” and “How often do you feel isolated from others?” A mean score was calculated across the three items.

Second, *loneliness in private and study contexts* was measured using a 9-item scale. The items were, e.g., “When you think about your personal contacts and relationships right now, how often do you feel lonely in those situations?” “If you feel lonely: How much does that feeling of loneliness weigh on you?” “When you feel lonely: How often do you talk to someone about it?” “When you think about your university years and the people you met there, how often do you feel lonely there?” and “What about you—when you feel lonely while you’re in university, how much does that feeling of loneliness weigh on you?” Response options ranged from 1 (“often”) to 4 (“never”), or 1 (“very strongly) to 4 (“not at all”), where higher scores reflect lower levels of loneliness. This scale was piloted and used in German before but has not yet been validated.

### Social participation

Social participation at the university was assessed with the following items “I enjoy studying at the university,” “I don’t really care whether I am studying at the university or another one (reversed)”, “I am engaged with issues related to my studies (e.g., student representative)”, “When I study, I consciously only do what is really asked of me” (reversed), and “I am involved at my university (e.g., Undergraduate Student Government, USG; Graduate Student Association, GSA)”. Answers could be given from 1 = “does not apply at all” to 5 = “fully applies.” A comparable questionnaire is the University Student Engagement Inventory (Assunção et al., 2020). It has 3 dimensions (behavioral engagement, emotional engagement, cognitive engagement), each measured by 5 items, and emotional engagement concepts overlap with Cronbach’s α = 0.88 (Assunção et al., 2020).

### Self-efficacy

General self-efficacy was measured using the 10-item scale by Schwarzer and Jerusalem (1995). This scale was validated (Cronbach’s α = 0.80 + in Germany; Cronbach’s α = 0.76–0.90 across other countries). The items were worded “I can always manage to solve difficult problems if I try hard enough,” “If someone opposes me, I can find the means and ways to get what I want,” “It is easy for me to stick to my aims and accomplish my goals,” “I am confident that I could deal efficiently with unexpected events,” “Thanks to my resourcefulness, I know how to handle unforeseen situations,” “I can solve most problems if I invest the necessary effort,” “I can remain calm when facing difficulties because I can rely on my coping abilities,” “When I am confronted with a problem, I can usually find several solutions.” “If I am in trouble, I can usually think of a solution.” and “I can usually handle whatever comes my way.” Items were rated from 1 (“not at all true”) to 4 (“exactly true”), and a mean score was computed, with higher values indicating greater self-efficacy.

### Study satisfaction

Overall satisfaction with the study situation was assessed with a single item (Westermann et al., 2018), from the validated scale (the 9-item scale has Cronbach’s α = 0.71–0.91). The item is worded, “How satisfied are you with your university experience in general?” Answers could be rated from 0 (“very dissatisfied”) to 100 (“completely satisfied”), with higher scores indicating stronger satisfaction.

### Study conditions

Study-related contextual factors were assessed using a validated instrument (Lehnchen et al., 2025). It was capturing multiple dimensions of academic and institutional conditions. Based on factor analysis in the present sample, the items were aggregated into six factors: Factor1 “academic support,” Factor2 “healthy options,” Factor3 “help with time conflicts,” Factor4 “advisory service,” Factor5 “good communication,” and Factor6 “supporting breaks and with family duties,” with higher scores indicating that students perceived this contextual factor as more supportive. The wording of the items was, for instance, “The way my studies are organized during the examination period minimizes stress (e.g., by sufficient time between examinations, the possibility of a second examination date).” “Appropriate breaks are planned between the courses.” and “Overall, people experience a culture of open communication at the university.”

### Health behaviors

*Physical activity* was measured as the number of days per week (0–7) participants engaged in moderate or vigorous activity. The item stem was worded “On how many days in the last 7 days have you performed the following physical activities?” with the item “…Strenuous physical activity (requires a lot of breathing, e.g., fast cycling, training with weights) for at least 20 min” and “…Moderate physical activity (requires more breathing, e.g., quiet cycling or cleaning) for at least 30 min.” Additionally, *nutritional behavior* was assessed with a single item on daily fruit and vegetable consumption with ordinal response categories. The item was “How many portions of fruit and vegetables do you eat on average every day (1 portion = 1 medium-sized piece of fruit, a small bowl of salad, ½ cup of dried fruit)?” This could be answered with the answering options (0 = No fruit or vegetables, 1 = 1–2 portions, 2 = 3–4 portions, and 3 = 5 portions or more). All items were validated before with good measurement qualities (Lippke et al., 2009).

### Subjective health status

Self-rated health was measured using a single item from the German version of the SF-36 health survey (Morfeld et al., 2011). The item was worded “How would you describe your overall health status?” with responses ranging from 1 (“very good”) to 5 (“very poor”). Higher scores indicate poorer perceived health.

### Sociodemographic variables

Sociodemographic characteristics were assessed using seven items. Gender was recoded into a categorical variable. The study program was assessed using a single item that distinguished between bachelor’s and master’s programs. Additional variables were included such as age and semester.

### Statistical analyses

All *statistical analyses* were conducted using IBM SPSS Statistics. Initially, descriptive statistics were computed for all study variables, including frequencies, means (*M*), standard deviations (SD), and measures of distribution (e.g., skewness and kurtosis). Before inferential analyses, we evaluated the distribution of continuous variables using descriptive indices and visual inspection of histograms and Q-Q plots to assess the assumption of normality. Variables included in the parametric analyses showed approximately normal distributions and were therefore analyzed using parametric statistical procedures.

To examine associations between key variables, Pearson product–moment correlation coefficients (*r*) were calculated. Building on these bivariate analyses, multiple regression analyses were conducted to examine the predictive relationships between the independent and dependent variables while controlling for relevant covariates. In addition, multivariate analyses of variance (MANOVA) were conducted to assess group differences across multiple dependent variables simultaneously. Where assumptions for parametric testing were not met, non-parametric alternatives were applied as appropriate. Receiver operating characteristic (ROC) analyses were also conducted to evaluate the discriminatory power and diagnostic accuracy of selected variables. All statistical tests were conducted using a significance level of α = 0.05. Effect sizes and the strength of observed relationships were interpreted in accordance with established benchmarks (Cohen, 1992), allowing for a standardized evaluation of practical significance.

## Results

*Research Question 1:* (a) How lonely are students feeling relating to studying/working from home, their contacts at the university, and in private life, etc., and (b) if students report engaging in specific activities to cope with loneliness, do they differ in their loneliness from those not engaging in these activities?

Students reported a mean loneliness score of 2.76 (SD = 1.06; see Table 1). If the loneliness mean score was 3 or higher, students were categorized as rather lonely (*n* = 122, 41.6%), versus *n* = 171 (58.4%) categorized as rather well, not lonely/well connected.

**Table 1:** Intercorrelations and prevalences.

Variables	0	1	2	3	4	5	6	7	8	9	10	11	12	13	14	15	16	17	18
0 PA strenuous																			
1 PA moderate	0.60^**^																		
2 F&V	0.24^**^	0.29^**^																	
3 SE	0.21^**^	0.28^**^	0.06																
4 Health Literacy Self	0.16^*^	0.19^**^	0.11	0.59^**^															
5 HL R	0.23^**^	0.14^*^	0.26^**^	0.55^**^	0.74^**^														
6 Academic support	0.01	0.02	0.06	0.15^*^	0.20^**^	0.18^**^													
7 Healthy options	0.07	0.06	0.03	0.22^**^	0.23^**^	0.16^*^	0.40^**^												
8 Help with time conflicts	0.13^*^	0.17^**^	−0.02	0.14^*^	0.09	0.12	0.02	0.02											
9 Advisory service	0.14^*^	0.09	0.03	0.09	0.08	0.12	0.06	0.11	0.54^**^										
10 Good communication	−0.01	−0.08	0.16^*^	0.14^*^	0.17^*^	0.23^**^	0.35^**^	0.34^**^	−0.08	0.12									
11 Supporting breaks and with family duties	0.01	0.01	−0.13^*^	0.01	0.01	−0.01	0.34^**^	0.24^**^	0.15^*^	0.18^**^	0.18^**^								
12 UCLA_LS	−0.09	−0.05	−0.03	−0.17^**^	−0.16	−0.18^**^	−0.12	−0.29^**^	−0.24^**^	−0.28^**^	−0.16^*^	−0.03							
13 PHQ4_Depr	−0.14^*^	−0.07	−0.03	−0.20^**^	−0.27^**^	−0.31^**^	−0.12	−0.10	−0.31^**^	−0.25^**^	−0.12	−0.13^*^	0.53^**^						
14 PHQ4_Anx	−0.11	−0.04	0.06	−0.22^**^	−0.16^*^	−0.18^**^	−0.15^*^	−0.15^*^	−0.38^**^	−0.32^**^	−0.07	−0.20^**^	0.51^**^	0.69^**^					
15 Stress	−0.19^**^	−0.09	0.03	−0.13^*^	−0.10	−0.11	−0.08	−0.08	−0.45^**^	−0.36^**^	−0.04	−0.24^**^	0.42^**^	0.50^**^	0.65^**^				
16 Study satisfaction	0.07	−0.01	0.01	0.24^**^	0.15^*^	0.18^**^	0.29^**^	0.19^**^	0.18^**^	0.34^**^	0.35^**^	0.28^**^	−0.27^**^	−0.31^**^	−0.28^**^	0.26^**^			
17 Subjective health	−0.31^**^	−0.26^**^	−0.12^*^	−0.21^**^	−0.17^**^	−0.23^**^	−0.08	−0.12	−0.25^**^	−0.19^**^	−0.14^*^	−0.13^*^	0.28^**^	0.42^**^	0.36^**^	0.37^**^	−0.19^**^		
18 Social participation	0.04	0.02	0.14	0.32^**^	0.13	0.19^*^	0.26^**^	0.13	0.16	0.10	0.30^**^	0.14	−0.29^**^	−0.21^*^	−0.06	−0.20^*^	0.46^**^	−0.20^*^	
*M (SD)* *n*	2.40 (1.99) 288	3.21 (2.01) 289	2.39 (0.68) 288	2.95 (0.61) 229	2.94 (0.70) 229	3.06 (0.75) 229	3.03 (0.81) 255	3.12 (0.96) 238	2.58 (0.83) 255	2.69 (0.75) 255	3.22 (1.06) 240	3.18 (0.98) 249	2.76 (1.06) 277	2.25 (1.68) 280	2.61 (1.77) 279	2.40 (0.90) 280	57.37 (25.79) 227	2.42 (0.88) 292	2.92 (0.71) 132

0 = strenuous physical activity: On how many days in the last 7 days have you performed the following physical activities? Strenuous physical activity (requires a lot of breathing, e.g., fast cycling, training with weights) for at least 20 min; 1/ PA moderate = physical activity: On how many days in the last 7 days have you done the following physical activities? Moderate physical activity (requires more breathing, e.g., quiet cycling or cleaning) for at least 30 min. 2/ F&V = Portions of fruit and vegetables on average per every day, with categories (0 = No fruit or vegetables, 1 = 1–2 portions, 2 = 3–4 portions, and 3 = 5 portions or more)?; 3/ SE = Self-Efficacy; 5/ HL R = Health Literacy Responsibility (1–4). Bivariate correlations, means (M), standard deviations (SD) and sample size (n) are reported. *p < 0.05; **p < 0.01.

To assess the construct validity of the UCLA-LS and the reliability of the loneliness categorization into dichotomized high loneliness vs. low, we tested the contingency of two groups and four related items.

First, the two groups were analyzed by how often students *felt lonely when they thought about their private contacts and relationships* (see Appendix Table A1). Those categorized as rather not lonely in general reported rarely or never feeling lonely with their private contacts (75.3%). In comparison, those categorized as rather lonely in general reported this often or sometimes (77.7%). A clear and significant correlation between the two variables was found, with χ^2^(3) = 85.79, *p* < 0.01; Contingency Coefficient = 0.49, *p* < 0.01. Second, regarding the extent to which *that feeling of loneliness weighed on them*, there was a clear association with the UCLA-LS measure: Those who felt lonely were more likely to report being affected (49.1%) than those who did not (16.2%; see Appendix Table A2). This was statistically significant, χ^2^(3) = 30.38, *p* < 0.01; Contingency Coefficient = 0.35; *p* < 0.01.

Third, when asked how often students would *feel lonely in their studies and with their contacts at the university*, clear differences emerged: non-lonely students reported 58.5% rarely or never, and generally lonely students reported 79.5% often or sometimes (see Appendix Table A3). This was statistically significant with χ^2^(3) = 43.95, *p* < 0.01; Contingency Coefficient = 0.36; *p* < 0.01.

Moreover, when asked what it would be like for them when *feeling lonely during studies and how much this feeling of loneliness would weigh on them*, also clear differences transpired; non-lonely students answered with 43.9% less strong or as good as not at all, and generally lonely students reported with 61.5% very strong or rather strong (see Appendix Table A4). This was statistically significant with χ^2^(3) = 22.28, *p* < 0.01; Contingency Coefficient = 0.276; *p* < 0.01.

To test the *sensitivity and specificity* of the grouped UCLA-LS measure with the above items, we calculated the ROC curve (see Figure 1).

**Figure 1:**
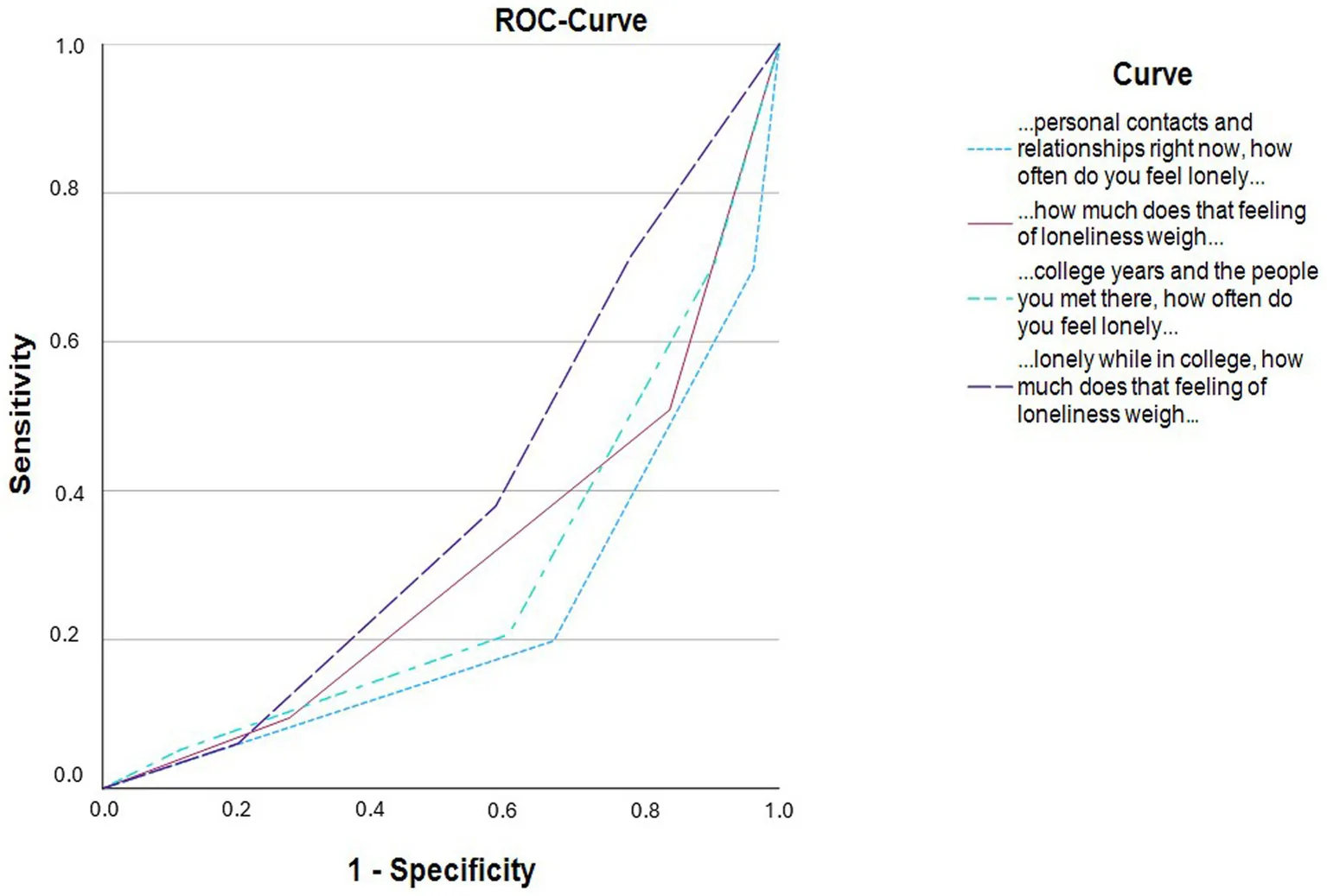
ROC curve of the high/low UCLA-LS with the four alternative loneliness measures.

The Area Under the Curve (AUC) was significant for all four measures (see Appendix Table A5) and showed good sensitivity and specificity (Appendix Table A6). This supported the UCLA-LS as a very good measure and the reliability of grouping into high and low loneliness. Accordingly, both the UCLA-LS and the dichotomized variable appear reliable for further use.

Finally, to check whether there are interrelations with *working or studying from home*, no differences were revealed, with generally 46.4% of students not working/studying from home, and 53.6% working/studying from home, and about equal numbers of students feeling lonely (Appendix Table A7; χ^2^(1) = 0.11; Contingency Coefficient = 0.02; *p* = 0.74).

Furthermore, we asked whether students *would talk to someone about their loneliness*. There were no differences between the groups, with 7.6% answering always, 31.4% sometimes, 38.1% rarely, and 22.9% never (see Appendix Table A8). This was not significant with χ^2^(3) = 4.09; Contingency Coefficient = 0.13; *p* = 0.25.

To understand if students report engaging in *specific activities to cope with loneliness*, differ in their loneliness from those not engaging in these activities (such as talking to friends, family, professionals or being in solitude, doing sports, watch TV, go into the nature, do gardening, cook, read, look for others on the Internet or social media, snack, shop etc.) we asked about different behavioral domains (see Table 2), to which they could agree or not.

**Table 2:** Mean loneliness of students agreeing to perform the behavioral domains and statistical test of differences.

	Loneliness	*M (SD)*	*F*(1, 275) =	Eta^2^=
Talk to friends or acquaintances	No	2.65 (1.11)	2.61	0.01
	Yes	2.89 (0.99)		
Talk to family or relatives	No	2.73 (1.10)	0.49	<0.01
	Yes	2.83 (0.97)		
Talk to a doctor or therapist	No	2.67 (1.04)	21.59**	0.07
	Yes	3.63 (0.86)		
Talk to a professional through telephone or online counseling, e.g., telephone counseling	No	2.74 (1.07)	3.18	0.01
	Yes	3.42 (0.64)		
Talk to other affected persons on the Internet, e.g., via forums. blogs or social media	No	2.74 (1.06)	2.11	0.01
	Yes	3.19 (1.01)		
Do things on one’s own and go to the cinema, theater, or an exhibition	No	2.72 (1.05)	1.31	0.05
	Yes	2.89 (1.10)		
Do sport	No	2.73 (1.12)	0.45	<0.01
	Yes	2.82 (0.93)		
Watch TV or stream a series or movie	No	2.50 (1.07)	14.64**	0.05
	Yes	2.98 (1.01)		
Go for a walk or into nature	No	2.63 (1.12)	5.23*	0.02
	Yes	2.92 (0.96)		
Work in the garden or look after my balcony	No	2.78 (1.08)	0.85	<0.01
	Yes	2.54 (0.80)		
Cook or bake something	No	2.69 (1.08)	3.44	0.01
	Yes	2.95 (0.99)		
Read	No	2.72 (1.07)	0.83	<0.01
	Yes	2.84 (1.05)		
Look for contact with others on the Internet	No	2.67 (1.04)	9.57**	0.03
	Yes	3.20 (1.07)		
Pass the time on the Internet, e.g., on social media	No	2.51 (1.08)	15.40**	0.05
	Yes	2.99 (0.99)		
Take care of one's household or tidy up	No	2.61 (1.07)	14.04**	0.05
	Yes	3.12 (0.96)		
Play games or gamble	No	2.78 (1.10)	0.29	<0.01
	Yes	2.70 (0.89)		
Listen to something, e.g., music, a radio play, or a podcast	No	2.56 (1.06)	11.98**	0.04
	Yes	2.99 (1.02)		
Drink a glass of wine or beer	No	2.71 (1.04)	6.06**	0.02
	Yes	3.24 (1.13)		
Snack or eat something.	No	2.61 (1.08)	10.72**	0.04
	Yes	3.03 (0.97)		
Go shopping and treat oneself to something nice	No	2.69 (1.02)	4.52*	0.02
	Yes	3.02 (1.16)		
Smoke cigarettes. e-cigarettes or shisha	No	2.76 (1.07)	0.02	<0.01
	Yes	2.80 (0.87)		
Smoke a joint	No	2.74 (1.08)	1.27	0.01
	Yes	3.07 (0.71)		

*p < 0.05; **p < 0.01.

Significant group differences were found in 10 behaviors, particularly in talking to a doctor, watching TV or streaming, going for a walk in nature, looking for contacts with others on the Internet, passing the time on the Internet or social media, taking care of the household and tidying up, listening to something, consuming alcohol, eating, and shopping. In contrast, no differences were found in 12 additional behaviors. However, the general trend was-even if not statistically significant-that those performing the behaviors to cope with loneliness also reported more reasons for it, i.e., higher loneliness, with just one exception: those playing games or gambling reported about the same level of loneliness as those not doing this.

*Research Question 2:* How healthy are students’ lifestyles, and what role does loneliness play in this (in comparison to self-efficacy, health literacy, context, and socio-demographic factors)?

We used a stepwise logistic regression to predict whether students were performing a healthy lifestyle (*n* = 149, 50.9%) versus not meeting the criteria for a healthy lifestyle (*n* = 144, 49.1%; Table 3).

**Table 3:** Logistic regression predicting a healthy lifestyle.

	Model 1			Model 2			Model 3		
	OR	95% CI LL	95% CI UL	OR	95% CI LL	95% CI UL	OR	95% CI LL	95% CI UL
Gender	1.00	0.99	1.00	1.00	0.99	1.00	1.00	0.99	1.00
Semester truncated to 7 S	1.04	0.90	1.21	1.04	0.89	1.21	1.05	0.90	1.24
Bachelor/Master	1.16	0.55	2.45	1.01	0.45	2.24	0.92	0.40	2.11
Self-efficacy	2.40**	1.44	4.01	1.61	0.87	2.98	1.76	0.92	3.39
Health literacy self				1.01	0.53	1.91	1.00	0.51	1.96
Health literacy responsibility				1.78*	1.00	3.18	1.75	0.95	3.24
Academic support				1.07	0.70	1.62	1.19	0.77	1.85
Healthy options				1.08	0.76	1.51	1.07	0.75	1.53
Help with time conflicts				1.16	0.75	1.80	1.15	0.71	1.86
Advisory service				1.15	0.72	1.82	1.27	0.78	2.09
Good communication				0.93	0.68	1.26	0.97	0.70	1.34
Supporting breaks and with family duties				0.96	0.70	1.32	0.98	0.70	1.38
UCLA_LS							1.12	0.79	1.58
PHQ4_Depr							1.04	0.79	1.37
PHQ4_Anx							1.02	0.78	1.35
Stress							1.15	0.70	1.89
Study satisfaction							0.99	0.97	1.00
Subjective health							0.52**	0.34	0.80
*R* ^2^	0.08			0.13			0.21		

In the first step, self-efficacy significantly predicted a healthy lifestyle (OR = 2.40) but explained only 8% of the variance. In the second step, health literacy responsibility was significant (OR = 1.78), while self-efficacy was no longer significant, and it explained 13% of the variance. In the third step, 21% of the variance was explained, mainly by subjective health, with those reporting poor health status being about 50% less likely to engage in a healthy lifestyle. *p < 0.05; **p < 0.01.

*Research Question 3:* To what extent do health psychological factors (lifestyle, self-efficacy, health literacy, context, and mental health factors) help students to perform social participation in their university over and above loneliness?

To better understand the role of loneliness and whether it could function as a pressure to change, we also tested differences regarding loneliness and lifestyle with a MANOVA and the interaction (Appendix Table A9). Validating the analyses above and the correlational findings in Table 1, it transpired that lonelier students not only participated less socially but were also less health-literate in terms of responsibility, perceived less academic and advisory support, had poorer communication, and were more stressed and less satisfied with their studies. While the other variables did not differ significantly with respect to loneliness, students who practiced a healthy lifestyle differed from those who did not in self-efficacy, health literacy, and subjective health—predicting social participation -which was significantly interrelated with loneliness in the MANOVA (Appendix Table A9).

In a trimmed stepwise regression analysis predicting social participation, loneliness was a significant predictor (β = −0.30) only when not controlling for the subsequent variables. While loneliness was significantly predicted by more stress and less study satisfaction (but not context), social participation was predicted by higher self-efficacy, more supportive context, and study satisfaction see Figure 2.

**Figure 2:**
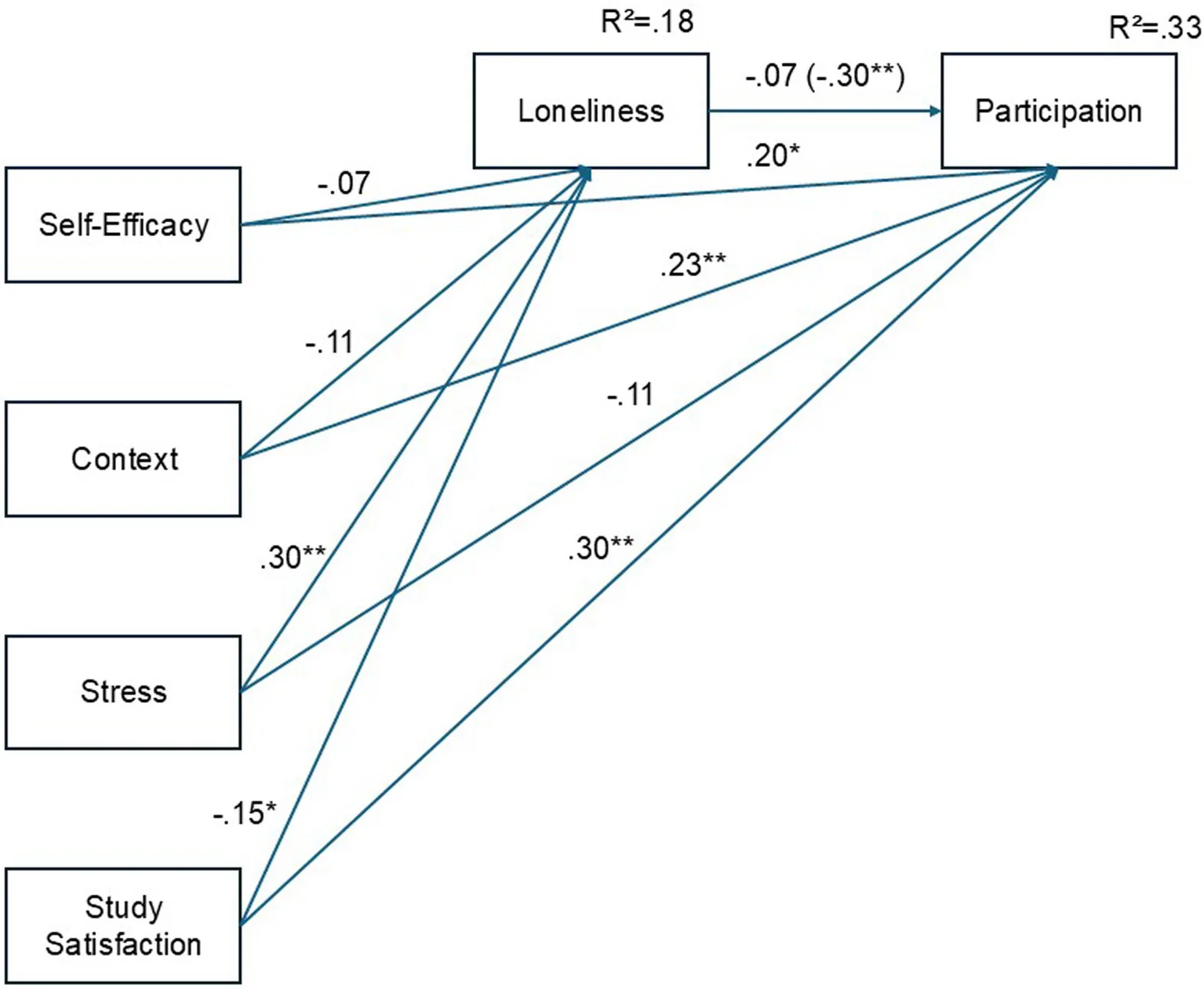
Prediction of loneliness and social participation by key variables. ** *p* < 0.01; * *p* < 0.05.

To test the interaction between stress and loneliness on social participation and examine non-linear interrelations, a regression analysis with curve prediction was conducted. It revealed that only among students with low loneliness there was a quadratic interrelation, leaving no variance to explain for the linear term, *F*(1, 76)_linear_ = 2.24; *R*^2^ = 0.03, not significant (Figure 3).

**Figure 3:**
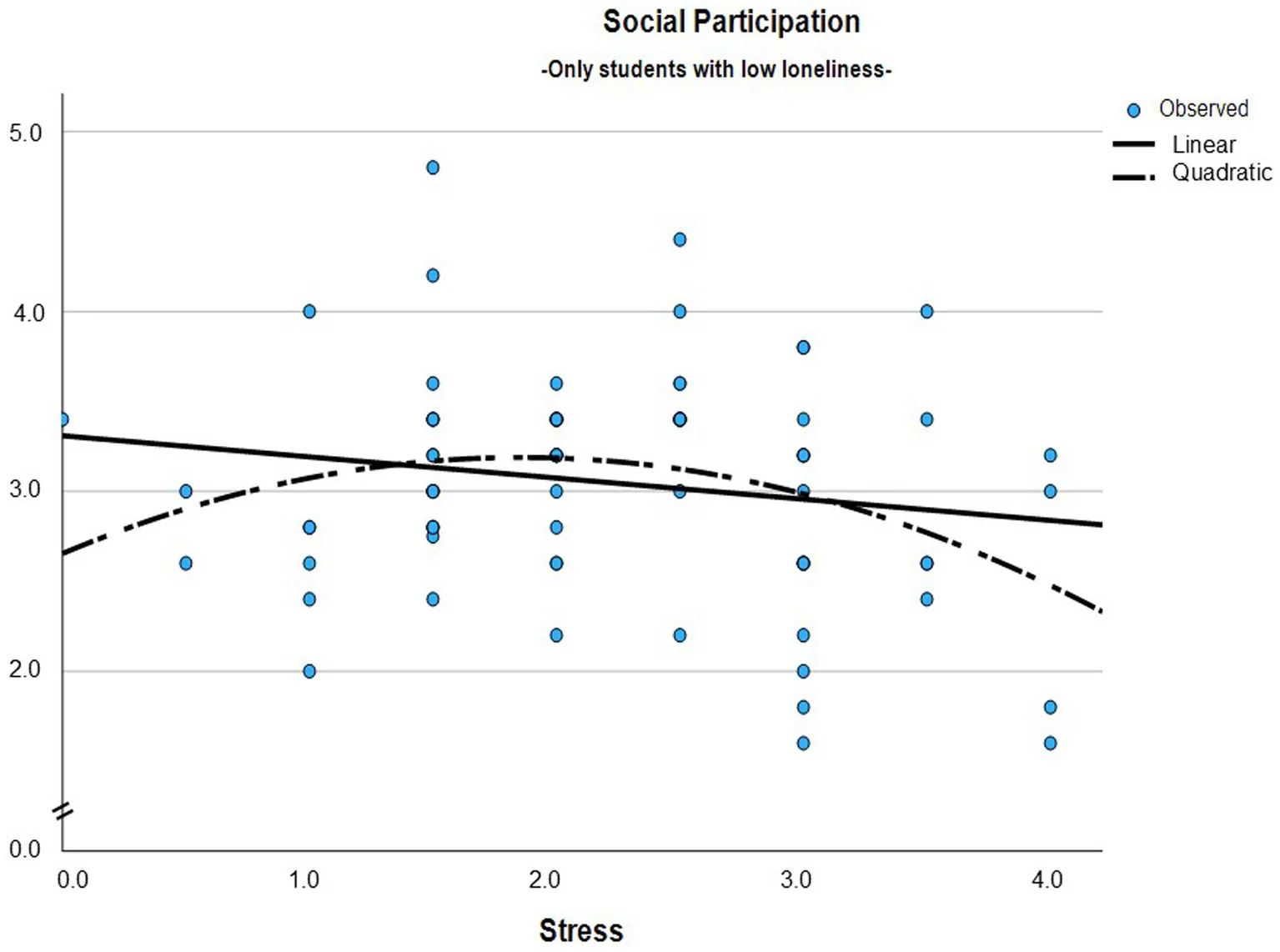
Non-linear interrelation of stress and social participation in students with low loneliness.

The quadratic term was *F*(2, 75)*_quadratic_* = 3.62; *R*^2^ = 0.09, *p* = 0.03, with parameters b1 = 0.58 and b2 = −0.16, which indicates that, firstly, more stress seems to enable more social participation, but from around a mean score of 2 on, the likelihood for social participation decreases—but this is only the case if students feel well connected. While the descriptive data appear similar, the differences were not statistically significant for students who felt rather lonely.

## Discussion

The present community case study included 373 students from a single community, i.e., an exemplary higher education institution (HEI). The study aimed to examine the prevalence and correlates of health and loneliness among university students, associated behavioral patterns, and the role of psychological factors in shaping lifestyle and social participation (Hysing et al., 2020; Migliorini et al., 2026). Several key findings emerged that contribute to a more nuanced understanding of student mental and physical health in higher education contexts: *Loneliness* was highly prevalent in the sample, with over 40% of students classified as rather lonely. This proportion is notable and aligns with growing evidence that loneliness represents a substantial health concern among university populations (Buecker et al., 2021). The consistency across different loneliness indicators, as well as the strong associations between the UCLA-LS and alternative measures, supports the classification’s robustness and validity. Importantly, loneliness was not confined to a single life domain but was reflected in both private and academic contexts (Lippke and Pappa, 2025a,b).

Students categorized as lonely reported more frequent feelings of social disconnection, perceived stronger negative impacts of loneliness on their studies, and experienced these feelings as more burdensome, as also shown in other studies (Homer et al., 2026; Terry and Branham, 2025). However, no association was found between loneliness and studying or working from home, suggesting that loneliness may depend less on physical study settings and more on subjective perceptions of social participation, which can be fostered even when students study from home. Also, no differences were observed in behaviors potentially coping with loneliness and mean loneliness levels regarding talking to others about their loneliness in general. Only talking to professionals was related to higher loneliness. This demonstrates that loneliness is not a general characteristic but rather that, when people feel lonely, they may find professional help or engage in specific behaviors, such as distracting themselves by watching TV, films or online content; spending time in nature or listening to music; cleaning the household; treating themselves to a glass of wine or beer; snacking or shopping; seeking resonance with themselves; or looking for contact with others on the Internet.

While some of these behaviors carry the risk of (too much) unhealthy consumption, we can summarize that students who performed the other 12 of the 22 behaviors, compared with those who did not, were not statistically lonelier. This may indicate that students successfully employed those behaviors to cope with their loneliness, and these behaviors provide preventive potential (Homer et al., 2026). The pattern suggests that loneliness is not associated with a uniform coping strategy but rather with a heterogeneous set of behaviors, possibly reflecting attempts to regulate negative affect (Ellard et al., 2023). Notably, no differences were found in core health behaviors such as sports, gardening, reading, or smoking, indicating that loneliness may be more strongly linked to everyday coping and leisure behaviors than to structured health routines.

Regarding *healthy lifestyles*, the results highlight the importance of psychological resources in accordance with theoretical assumptions: Self-efficacy initially emerged as a strong predictor of health behaviors, consistent with established theories (Cacioppo and Cacioppo, 2018; Lippke and DeSmet, 2025). However, its effect diminished if health literacy—particularly the responsibility component—was consistently included, suggesting overlapping or mediating mechanisms (Södel et al., 2025). In the final model, subjective health status was the most robust predictor, indicating a close interplay between perceived health and health-related behavior. Loneliness, in contrast, did not directly predict a healthy lifestyle when controlling for the other factors, suggesting that its impact may be more indirect.

Regarding *social participation*, loneliness was associated with lower levels of participation, consistent with previous findings demonstrating that loneliness can hinder participation in social and community activities (Cicognani et al., 2008; Youn et al., 2020). However, this relationship weakened if additional variables such as self-efficacy, perceived communication quality at the university, and study satisfaction were considered. This finding underscores the importance of psychological and contextual resources in facilitating student engagement. In particular, higher self-efficacy and supportive institutional communication appear to play a central role in enabling social participation, potentially buffering the negative effects of loneliness (Cicognani et al., 2008). These results suggest that loneliness alone may not directly determine students’ social participation; rather, its impact appears to operate within a broader network of personal and environmental resources. This can help to strengthen belonging and prevent chronic loneliness. Accordingly, the ETL may serve as a guide for examining and improving social participation and institutional belonging, and with that jointly contribute to student health and well-being.

Further analyses revealed a more nuanced relationship involving stress. Given the strong association between stress and loneliness, moderation analyses indicated a significant non-linear effect of stress on social participation: Among students with low loneliness, moderate stress was associated with increased social participation, whereas higher stress levels were linked to reduced social participation (Knapp et al., 2024). This inverted U-shaped relationship is consistent with theoretical perspectives suggesting that moderate levels of activation or challenge can motivate engagement. At the same time, excessive stress may overwhelm coping resources and hinder involvement in social activities. However, this relationship was not statistically significant among students experiencing higher levels of loneliness. One possible explanation is that loneliness may impair the capacity to transform moderate stress into adaptive, engagement-promoting behavior. In this sense, loneliness could function as a barrier that disrupts otherwise beneficial motivational processes, leaving students more vulnerable to withdrawal and reduced social participation. Future research is needed to examine the mechanisms underlying this interaction and to determine whether interventions targeting loneliness, self-efficacy, or stress management can enhance students’ social engagement and overall well-being.

While the observed differences across behavioral items provide valuable insights, they should be interpreted with some caution. The sample size (*N* = 293), although adequate for exploratory analyses, is relatively modest given the large number of behavioral items examined (22 items). This imbalance may affect the stability of the estimates and increase the likelihood that some observed differences reflect sampling variability rather than robust underlying patterns. Furthermore, analyzing numerous behavioral indicators can complicate interpretation, as individual item-level effects may be sensitive to specific sample characteristics. Therefore, the findings should be viewed as indicative rather than definitive, and future research with larger samples would help confirm the observed patterns and strengthen the generalizability of the results.

As a *community case study*, these findings should be interpreted in light of the study design, the focus on a single exemplary HEI, and the use of self-reported measures. Overall, the findings suggest that loneliness among students is a multifaceted phenomenon embedded within a broader network of psychological, behavioral, and contextual factors. Interventions aiming to reduce loneliness and promote social participation and well-being should therefore not only target social participation directly. Instead, they should also strengthen individual resources such as self-efficacy, enhance health literacy, and improve institutional conditions, including communication and community support services.

## Conclusion

This community case study identified loneliness as a prevalent and multifaceted challenge among university students, affecting more than 40% of the sample and extending across both academic and private life domains. The findings indicate that loneliness is closely associated with students’ lower social participation and diminished feelings of belonging, and poorer psychological well-being rather than with study settings such as working from home. Although loneliness was linked to several specific coping behaviors and greater use of professional support services, coping responses appeared heterogeneous across students. Furthermore, healthy lifestyles and social participation were more strongly explained by psychological and contextual resources, particularly self-efficacy, health literacy, subjective health status, study satisfaction, and perceived quality of university communication. The results also point to a complex interplay between loneliness and stress, whereby loneliness may impede the transition from moderate stress to adaptive social engagement.

These findings have important implications for multiple stakeholders. For *higher education institutions and university leaders*, the results highlight the need to move beyond isolated mental health initiatives and adopt comprehensive strategies that foster social participation, belonging, and supportive campus environments. For *student support services*, including counseling, mentoring, and health promotion programs, the findings suggest that interventions should not only address loneliness directly but also strengthen students’ self-efficacy, health literacy, and access to social resources. For *academic staff and educators*, creating inclusive learning environments and facilitating meaningful social interactions may contribute to both student well-being and academic success. Finally, for *policymakers and public health practitioners*, the findings emphasize the importance of recognizing loneliness as a community health issue and investing in evidence-based approaches that promote social connectedness and reduce inequalities within increasingly diverse student populations.

Taken together, these findings underscore that loneliness should not be understood as an isolated individual experience but as part of a broader network of psychological, behavioral, and environmental factors. Strengthening opportunities for social participation and fostering a sense of belonging may therefore represent key pathways for improving student well-being and creating healthier university communities by means of improving self-efficacy, health literacy, and supportive institutional environments.

Given the exploratory and cross-sectional nature of this single-site case study and the relatively modest sample size relative to the number of behavioral indicators examined, future research should replicate these findings across larger and more diverse student populations. Longitudinal and intervention studies are also needed to clarify causal relationships and identify the most effective strategies for promoting belonging, reducing loneliness, and enhancing student health and well-being.

## Data Availability

The raw data supporting the conclusions of this article can be obtained from the first author.
