# Supplementary material for: Health behavior, loneliness and social participation among students in a higher education institution: a community case study providing insights into health psychology
Source: Front Psychol. 2026 Oct 12;17:1894036. doi: 10.3389/fpsyg.2026.1894036 (PMC13696492; doi:10.3389/fpsyg.2026.1894036)
Supplement: Supplementary file 1 [file Supplementary_file_1.docx]

# **Supplementary Material**

Appendix Table A1. Students Reporting Low vs. High Loneliness on the UCLA-LS by Perceived Loneliness Regarding Personal Contacts, *n* (%).

|  | | | When you think about your personal contacts and relationships right now, how often do you feel lonely in those situations? | | | | Total |
| --- | --- | --- | --- | --- | --- | --- | --- |
|  |  |  | Often | Sometimes | Rarely | Never |  |
| UCLA_LS_dichotomized | Low loneliness | *n* | 5 | 33 | 77 | 39 | 154 |
|  |  | % | 3.2% | 21.4% | 50.0% | 25.3% | 100.0% |
|  | High loneliness | *n* | 35 | 59 | 24 | 3 | 121 |
|  |  | % | 28.9% | 48.8% | 19.8% | 2.5% | 100.0% |
| Total | | *n* | 40 | 92 | 101 | 42 | 275 |
|  |  | % | 14.5% | 33.5% | 36.7% | 15.3% | 100.0% |

Appendix Table A2. Students Reporting Low vs. High Loneliness on the UCLA-LS by Perceived Loneliness Subjectively Weighted on Individuals with Private Contacts, *n* (%).

|  | | | If you feel lonely: How much does that feeling of loneliness weigh on you? | | | | Total |
| --- | --- | --- | --- | --- | --- | --- | --- |
|  |  |  | Very strongly | Rather strongly | To a lesser extent | Not at all |  |
| UCLA_LS_dichotomized | Low  loneliness | *n* | 6 | 11 | 59 | 29 | 105 |
|  |  | % | 5.7% | 10.5% | 56.2% | 27.6% | 100.0% |
|  | High loneliness | *n* | 20 | 37 | 48 | 11 | 116 |
|  |  | % | 17.2% | 31.9% | 41.4% | 9.5% | 100.0% |
| Total | | *n* | 26 | 48 | 107 | 40 | 221 |
|  |  | % | 11.8% | 21.7% | 48.4% | 18.1% | 100.0% |

Appendix Table A3. Students Reporting Low vs. High Loneliness on the UCLA-LS by Perceived Loneliness Regarding University Contacts, *n* (%).

|  | | | When you think about your college years and the people you met there, how often do you feel lonely there? | | | | Total | |
| --- | --- | --- | --- | --- | --- | --- | --- | --- |
|  |  |  | Often | Sometimes | Rarely | Never |  |  |
| UCLA_LS_dichotomized | Low loneliness | *n* | 33 | 38 | 61 | 39 | 171 | |
|  |  | % | 19.3% | 22.2% | 35.7% | 22.8% | 100.0% | |
|  | High loneliness | *n* | 39 | 58 | 19 | 6 | 122 | |
|  |  | % | 32.0% | 47.5% | 15.6% | 4.9% | 100.0% | |
| Total | | *n* | 72 | 96 | 80 | 45 | 293 | |
|  |  | % | 25.5% | 32.8% | 27.3% | 15.4% | | 100.0% |

Appendix Table A4. Students Reporting Low vs. High Loneliness on the UCLA-LS by Perceived Loneliness Subjectively Affecting Individuals in University Contacts, *n* (%).

|  | | | When you feel lonely while you’re in college, how much does that feeling of loneliness weigh on you? | | | | Total |
| --- | --- | --- | --- | --- | --- | --- | --- |
|  |  |  | Very strongly | Rather strongly | To a lesser extent | Not at all |  |
| UCLA_LS_dichotomized | Low  loneliness | *n* | 73 | 23 | 47 | 28 | 171 |
|  |  | % | 42.7% | 13.5% | 27.5% | 16.4% | 100.0% |
|  | High loneliness | *n* | 36 | 39 | 40 | 7 | 122 |
|  |  | % | 29.5% | 32.0% | 32.8% | 5.7% | 100.0% |
| Total | | *n* | 109 | 62 | 87 | 35 | 293 |
|  |  | % | 37.2% | 21.2% | 29.7% | 11.9% | 100.0% |

Appendix Table A5. Predictive Validity and Area Under the Curve (AUC) for Different Study Loneliness Items.

| Variables | Area | SE | Asymptotic Significance | Asymptotic 95% Confidence Interval | |
| --- | --- | --- | --- | --- | --- |
|  |  |  |  | Lower limit | Upper limit |
| When you think about your personal contacts and relationships right now, how often do you feel lonely in those situations? | .23 | .03 | <.01 | .17 | .29 |
| If you feel lonely: How much does that feeling of loneliness weigh on you? | .31 | .04 | <.01 | .24 | .37 |
| When you think about your college years and the people you met there, how often do you feel lonely there? | .29 | .04 | <.01 | .22 | .36 |
| When you feel lonely while you’re in college, how much does that feeling of loneliness weigh on you? | .39 | .04 | .01 | .31 | .46 |

Appendix Table A6. Coordinates of the ROC-Curve.

| Variables | Positive, when  larger or equal | Sensitivity | Specificity |
| --- | --- | --- | --- |
| When you think about your personal contacts and relationships right now, how often do you feel lonely in those situations? | 0.00 | 1.00 | 1.00 |
|  | 1.50 | 0.70 | 0.96 |
|  | 2.50 | 0.20 | 0.67 |
|  | 4.00 | <.01 | <.01 |
| If you feel lonely: How much does that feeling of loneliness weigh on you? | 0.00 | 1.00 | 1.00 |
|  | 1.50 | 0.83 | 0.94 |
|  | 2.50 | 0.51 | 0.84 |
|  | 3.50 | 0.10 | 0.28 |
|  | 5.00 | <.01 | <.01 |
| When you think about your college years and the people you met there, how often do you feel lonely there? | 0.00 | 1.00 | 1.00 |
|  | 1.50 | 0.71 | 0.91 |
|  | 2.50 | 0.21 | 0.60 |
|  | 3.50 | 0.05 | 0.11 |
|  | 5.00 | <.01 | <.01 |
| When you feel lonely while you’re in college, how much does that feeling of loneliness weigh on you? | 0.00 | 1.00 | 1.00 |
|  | 1.50 | 0.72 | 0.78 |
|  | 2.50 | 0.38 | 0.58 |
|  | 3.50 | 0.06 | 0.20 |
|  | 5.00 | <.01 | <.01 |

Appendix Table A7. Students Reporting Low vs. High Loneliness on the UCLA-LS by Studying/ Working from Home Frequency (No/Low vs. Often/Only), *n* (%).

|  | | | Studying/ working from home dichotomized | | Total |
| --- | --- | --- | --- | --- | --- |
|  |  |  | No home studying | Regular home studying |  |
| UCLA_LS_dichotomized | Low loneliness | *n* | 78 | 93 | 171 |
|  |  | % | 45.6% | 54.4% | 100.0% |
|  | High loneliness | *n* | 58 | 64 | 122 |
|  |  | % | 47.5% | 52.5% | 100.0% |
| Total | | *n* | 136 | 157 | 293 |
|  |  | % | 46.4% | 53.6% | 100.0% |

Appendix Table A8. Students Reporting Low vs. High Loneliness on the UCLA-LS by Communication Frequency About Loneliness, *n* (%).

|  | | When you feel lonely: How often do you talk to someone about it? | | | | | | Total |
| --- | --- | --- | --- | --- | --- | --- | --- | --- |
|  |  | Often | | Sometimes | Rarely | Never | |  |
| UCLA_LS_dichotomized | Low  loneliness | *n* | 7 | 38 | 34 | | 27 | 106 |
|  |  | % | 6.6% | 35.8% | 32.1% | | 25.5% | 100.0% |
|  | High loneliness | *n* | 10 | 32 | 51 | | 24 | 117 |
|  |  | % | 8.5% | 27.4% | 43.6% | | 20.5% | 100.0% |
| Total | | *n* | 17 | 70 | 85 | | 51 | 223 |
|  |  | % | 7.6% | 31.4% | 38.1% | | 22.9% | 100.0% |

Appendix Table A9. MANOVA: Means, Standard Deviations and One-Way Analyses of Variance.

| Measure | Low loneliness | | High loneliness | | *F_Loneliness_*  (1, 225) | η^2^ | *F_HealthyLifestyle_*  (1, 225) | η^2^ |  |  |
| --- | --- | --- | --- | --- | --- | --- | --- | --- | --- | --- |
|  | *M* | *SD* | *M* | *SD* |  |  |  |  |  |  |
| Participation | 3.07 | 0.63 | 2.69 | 0.82 | 7.06** | .06 | 0.31 | .01 |  |  |
| SE | 3.03 | 0.60 | 2.86 | 0.58 | 1.73 | .02 | 4.55* | .04 |  |  |
| HL S | 3.01 | 0.71 | 2.82 | 0.73 | 1.87 | .02 | 7.06** | .06 |  |  |
| HL R | 3.16 | 0.73 | 2.86 | 0.74 | 4.39* | .04 | 5.80* | .05 |  |  |
| Factor1 academic support | 3.19 | 0.70 | 2.84 | 0.85 | 4.63* | .04 | 1.83 | .02 |  |  |
| Factor2 healthy options | 3.27 | 0.90 | 2.95 | 0.98 | 3.08 | .03 | 1.51 | .01 |  |  |
| Factor3 help with time conflicts | 2.60 | 0.77 | 2.51 | 0.77 | 0.80 | .01 | 3.35 | .03 |  |  |
| Factor4 advisory service | 2.863 | 0.71 | 2.45 | 0.73 | 11.41** | .09 | 3.13 | .03 |  |  |
| Factor5 good communication | 3.479 | 1.00 | 2.766 | 1.04 | 11.58** | .09 | 0.39 | .01 |  |  |
| Factor6 supporting breaks and with family duties | 3.09 | 0.99 | 2.968 | 0.90 | 0.26 | .01 | 0.01 | .01 |  |  |
| Stress | 2.31 | 0.85 | 2.59 | 0.747 | 4.15* | .04 | 0.90 | .01 |  |  |
| Study satisfaction | 60.65 | 22.08 | 42.94 | 25.46 | 15.00** | .11 | 0.12 | .01 |  |  |
| Subjective health | 2.35 | 0.88 | 2.51 | 0.88 | 0.94 | .01 | 14.36** | .11 |  |  |

*Note*. ***p*<.01; **p*<.05.
